# Supplementary material for: Rab11A Functions as a Negative Regulator of Osteoclastogenesis through Dictating Lysosome-Induced Proteolysis of c-fms and RANK Surface Receptors
Source: Cells. 2020 Oct 31;9(11):2384. doi: 10.3390/cells9112384 (PMC7692573; doi:10.3390/cells9112384)
Supplement: Supplementary file 1 [file cells-09-02384-s001.pdf]

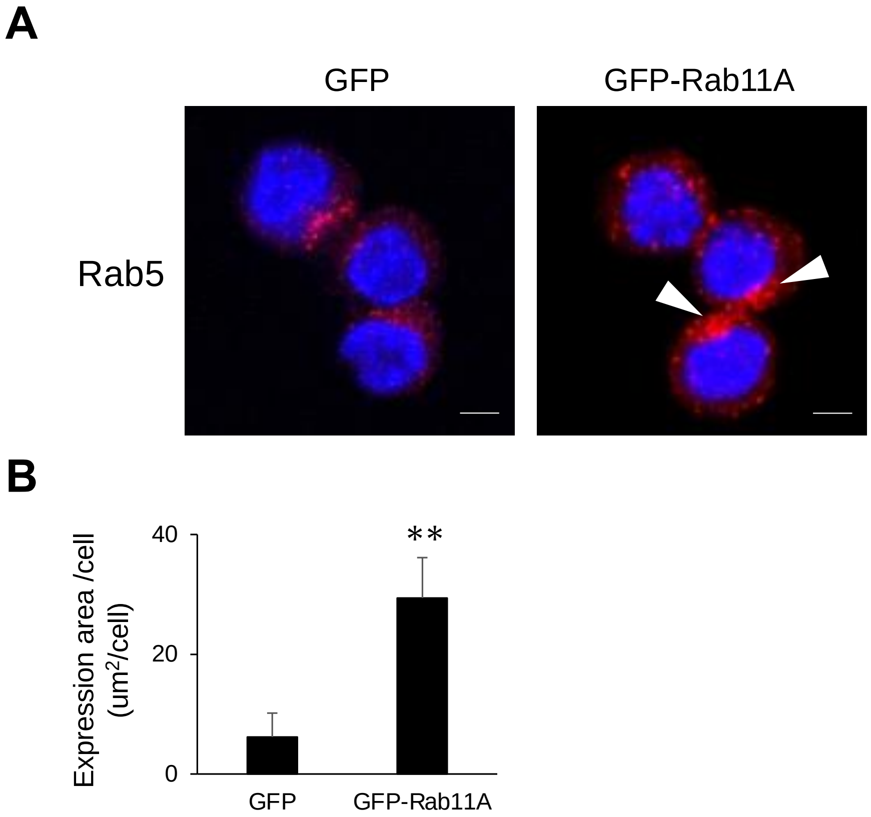

Figure S1. Comparison of Rab5 expression between GFP and GFP-Rab11A overexpress RAW-D cells. (A) Representative images of Rab5 expression (red) with the same exposure time. DNA was stained with DAPI (blue). Arrowheads indicated the positive region of Rab5. Scale bars: 5 μm. (B) Expression area divided the number of cells in the field was quantified and measured by ImageJ software. ** $p < 0.01$. Data are representative of three independent experiments.
